# InForm software: a semi-automated research tool to identify presumptive human hepatic progenitor cells, and other histological features of pathological significance

**DOI:** 10.1038/s41598-018-21757-4

**Published:** 2018-02-21

**Authors:** Anne S. Kramer, Bruce Latham, Luke A. Diepeveen, Lingjun Mou, Geoffrey J. Laurent, Caryn Elsegood, Laura Ochoa-Callejero, George C. Yeoh

**Affiliations:** 1 Harry Perkins Institute of Medical Research, QEII Medical Centre, Nedlands and Centre for Medical Research, The University of Western Australia, Crawley, WA Australia; 2 School of Molecular Sciences, The University of Western Australia, Crawley, WA Australia; 3 Centre for Cell Therapy and Regenerative Medicine, School of Biomedical Sciences, The University of Western Australia, Crawley, WA Australia; 4 PathWest Laboratory Medicine WA, Fiona Stanley Hospital, Murdoch, WA Australia; 5 WA Liver & Kidney Surgical Transplant Service, Sir Charles Gairdner Hospital, Nedlands, Australia; 6 School of Pharmacy and Biomedical Science, Curtin Health Innovation Research Institute, Curtin University, Bentley, WA Australia; 7 Angiogenesis group, Oncology Area, Centre for Biomedical Research of La Rioja, Logroño, Spain

## Abstract

Hepatic progenitor cells (HPCs) play an important regenerative role in acute and chronic liver pathologies. Liver disease research often necessitates the grading of disease severity, and pathologists’ reports are the current gold-standard for assessment. However, it is often impractical to recruit pathologists in large cohort studies. In this study we utilise PerkinElmer’s “InForm” software package to semi-automate the scoring of patient liver biopsies, and compare outputs to a pathologist’s assessment. We examined a cohort of eleven acute hepatitis samples and three non-alcoholic fatty liver disease (NAFLD) samples, stained with HPC markers (GCTM-5 and Pan Cytokeratin), an inflammatory marker (CD45), Sirius Red to detect collagen and haematoxylin/eosin for general histology. InForm was configured to identify presumptive HPCs, CD45^+ve^ inflammatory cells, areas of necrosis, fat and collagen deposition (p < 0.0001). Hepatitis samples were then evaluated both by a pathologist using the Ishak-Knodell scoring system, and by InForm through customised algorithms. Necroinflammation as evaluated by a pathologist, correlated with InForm outputs (r^2^ = 0.8192, p < 0.05). This study demonstrates that the InForm software package provides a useful tool for liver disease research, allowing rapid, and objective quantification of the presumptive HPCs and identifies histological features that assist with assessing liver disease severity, and potentially can facilitate diagnosis.

## Introduction

HPCs are a heterogeneous population, expressing immature and intermediate phenotypes of biliary and hepatic lineages^1^. Histologically, they are small ovoid cells with a high nuclear-to-cytoplasmic ratio. They are present in the healthy liver at low abundance, residing in the liver stem cell niche termed the “canals of Hering”^2,3^. The phenotype and distribution of HPCs vary according the liver pathophysiology and severity, and known markers including Pan Cytokeratin, CK19, NCAM and SOX-9 also stain cholangiocytes^4–7^. As such, the identification of HPCs is challenging, and a reliable method which is capable of identifying and quantifying HPCs of varying histological phenotypes is urgently required.

HPCs play an important role in repair, and have also been correlated with increased severity of chronic liver disease as well as development of hepatocellular carcinoma (HCC)^8–11^. When normal hepatocyte-mediated repair pathways are impaired, such as in severe acute or chronic liver disease, HPCs are activated to proliferate and differentiate towards hepatocytes and/or cholangiocytes to facilitate repair through regeneration^3,10,12^. The regulation of HPCs is complex and many cellular and extracellular partners have been identified, including stellate cells, macrophages, extracellular matrix and an intricate network of cytokines, adipokines and paracrine factors^5,13–15^. Together, the interactions of HPCs, the extracellular matrix and the associated inflammatory response has been termed “ductular reaction” in humans^4,16,17^, as the proliferation of HPCs is often of ductular phenotype^18,19^. The inflammatory response has a potent influence on HPC activation, and several pro-inflammatory cytokines have been shown to increase HPC proliferation^12,20–23^. The inflammatory environment contributes to tumour progression, and is associated with a higher risk of recurrence and poor prognosis of HCC, in part through enhanced proliferation of HPCs^24–26^. Like inflammation, the fibrotic response is closely correlated with the HPC proliferative response in many human liver pathologies including alcoholic- and non-alcoholic fatty liver disease, chronic hepatitis and genetic haemochromatosis^8,11,27^. Fibrogenesis is partly driven by HPCs through the release of pro-fibrotic factors which may, in turn, enhance HPC proliferation through positive feedback^4,19,28^. The effects of fatty deposits on HPCs has been less well characterised, but its importance is highlighted by the higher incidence of cirrhosis in obese patients, and the increased mortality of obese patients with HCC^29,30^. HPCs also produce cytokines termed ‘adipokines’, which have important roles in metabolic control, inflammation and tissue repair^31^. The levels of adipokines have been correlated with inflammation, fibrosis, and levels of fat and severity of NASH in several studies^31–33^.

Due to the intricate interactions of HPCs with inflammation, fibrosis and fat, HPC research often necessitates the assessment of these parameters. Traditionally, assessment by pathologists is the gold-standard approach, and many systems to semi-quantitatively score the necroinflammatory activity, fibrosis, and fat have been developed. The Ishak’s modification of Knodell’s “hepatic activity index” (referred to here as “Ishak-Knodell”) is a system designed for clinical assessment of chronic hepatitis^34^. The Ishak-Knodell system grades necroinflammatory activity using five categories; piecemeal necrosis, confluent necrosis, lobular necrosis and portal inflammation. The composite of these categories is then calculated to obtain the hepatic activity index (HAI), which reflects the necroinflammatory activity. Fibrosis is assessed using a separate staging category. The Ishak-Knodell, similar to other scoring systems, relies on the expertise of pathologists and thus is subjective by nature.

In this study, we have evaluated InForm as an alternative research tool to a pathologist’s assessment. We use custom designed algorithms to determine whether InForm can (i) identify and quantitate presumptive HPCs comparably to trained investigators (ii) identify histological features including inflammation, fibrosis and fat which are important in grading liver disease, and known to influence HPCs, and (iii) score the necroinflammatory activity in acute hepatitis patients consistent with a pathologist’s assessment using the Ishak-Knodell scale.

## Results

### InForm can be configured to quantitate and phenotype presumptive HPCs

**Figure 1:**
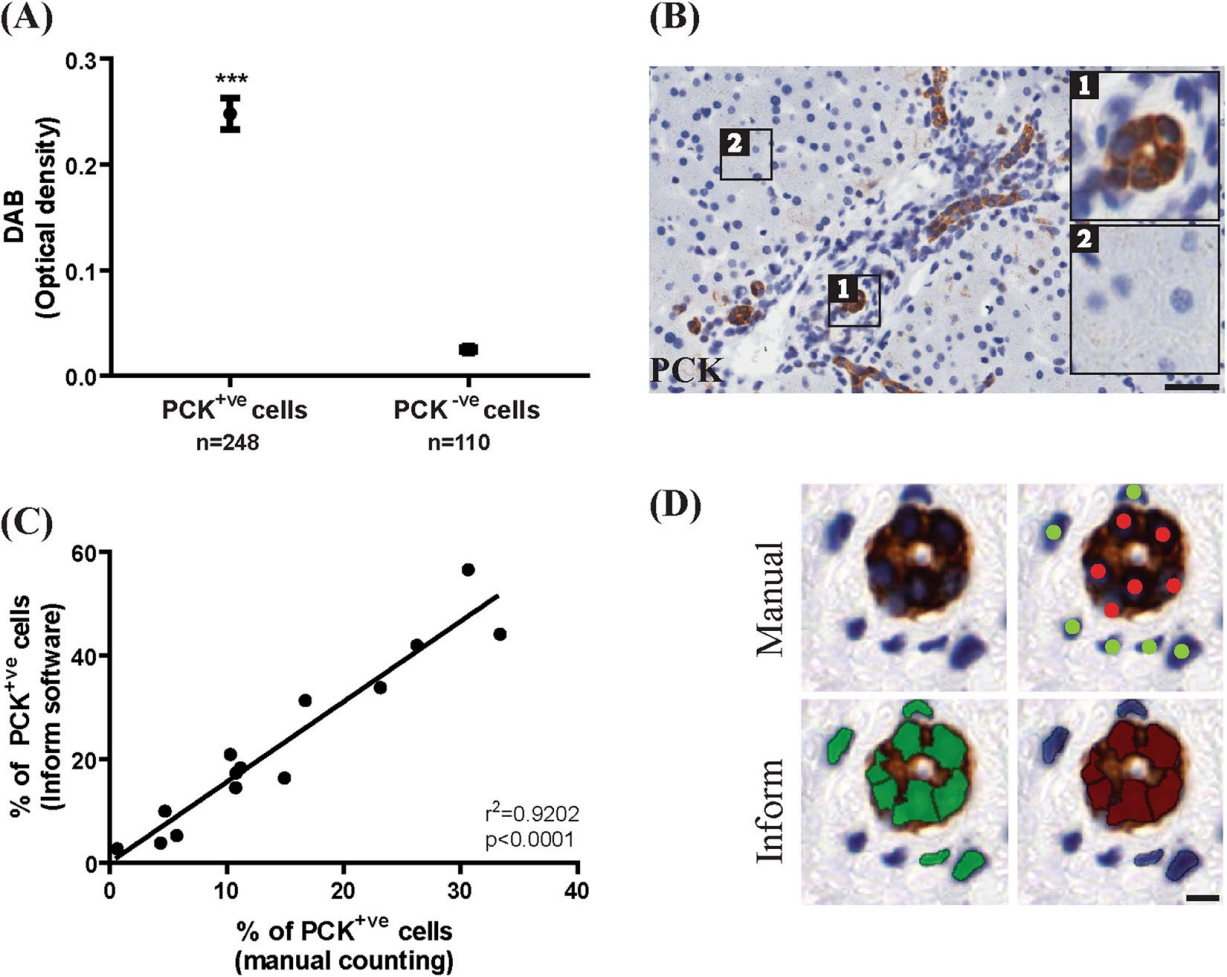
A custom algorithm was created to identify and quantitate PCK^+ve^ cells in immunohistochemically stained samples. (**A**) PCK^+ve^ and PCK^−ve^ cells were distinguished based on DAB levels. (**B**) Representative images of PCK^+ve^ cells (insert 1) and PCK^−ve^ cells (insert 2) are shown. (**C**) InForm output had a high correlation with manual counting (r^2^ = 0.9202, p < 0.0001, n = 14 FOVs). (**D**) Comparative images of cells counted manually (red indicates PCK^+ve^ cells, green indicates nuclei of cells that are PCK^−ve^), and with InForm (green indicates all nuclei counted by InForm, red indicates PCK^+ve^ nuclei). Scale bar 200 μm. ***p < 0.0001.

**Figure 2:**
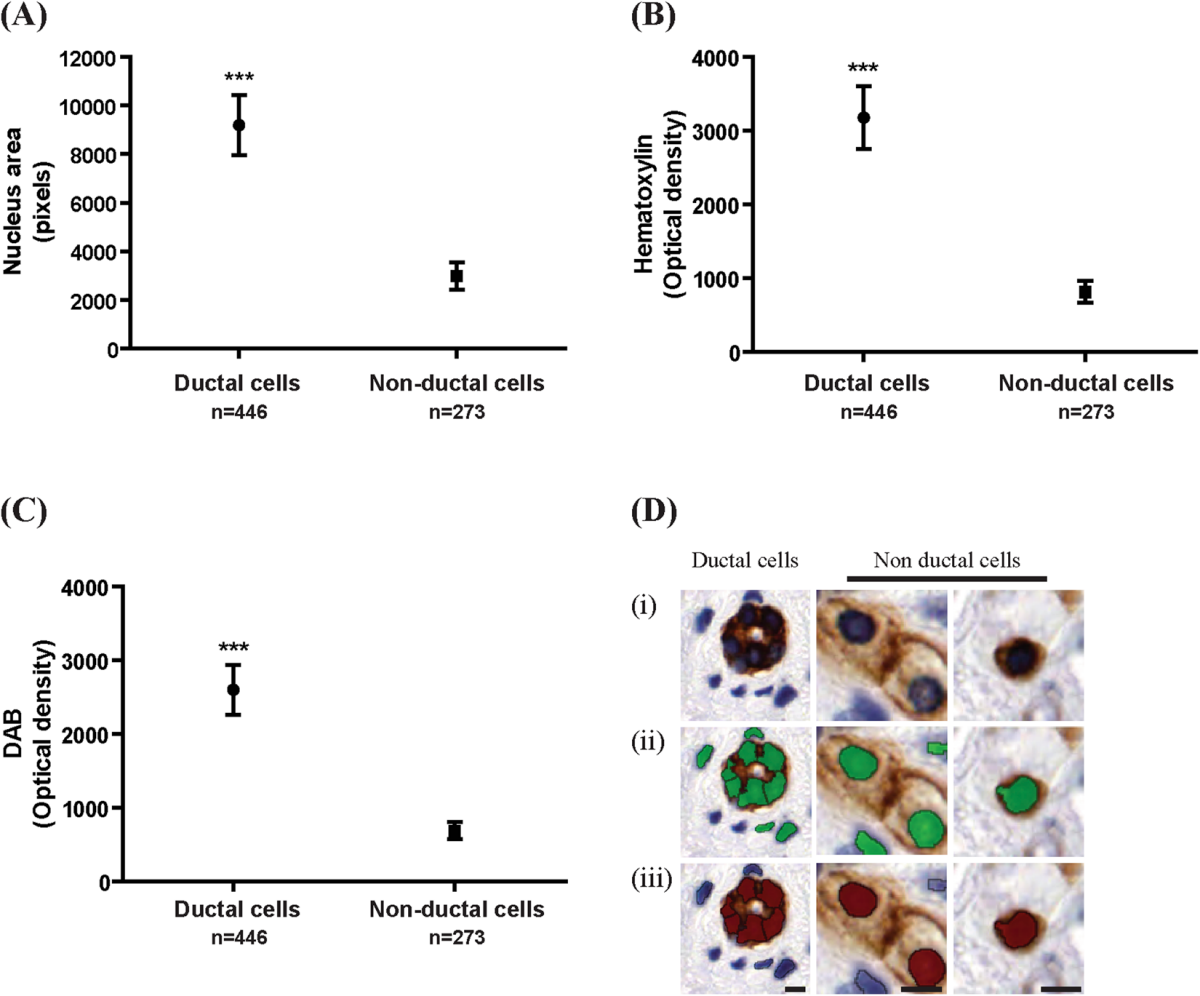
A custom algorithm was created to distinguish individual PCK^+ve^ ductal cells from PCK^+ve^ non-ductal cells in immunohistochemically stained samples. (**A**–**C**) Ductal cells have significantly higher nucleus area, and higher optical density of hematoxylin and DAB compared to non-ductal cells. (**D**) Representative images of PCK^+ve^ ductal and PCK^+ve^ non-ductal cells. (Di) DAB image. (Dii) Green cells indicates all nuclei counted by InForm. (Diii) Red cells indicates PCK^+ve^ cells counted by InForm (iii). Scale bar 20 μm. ***p < 0.0001.

**Figure 3:**
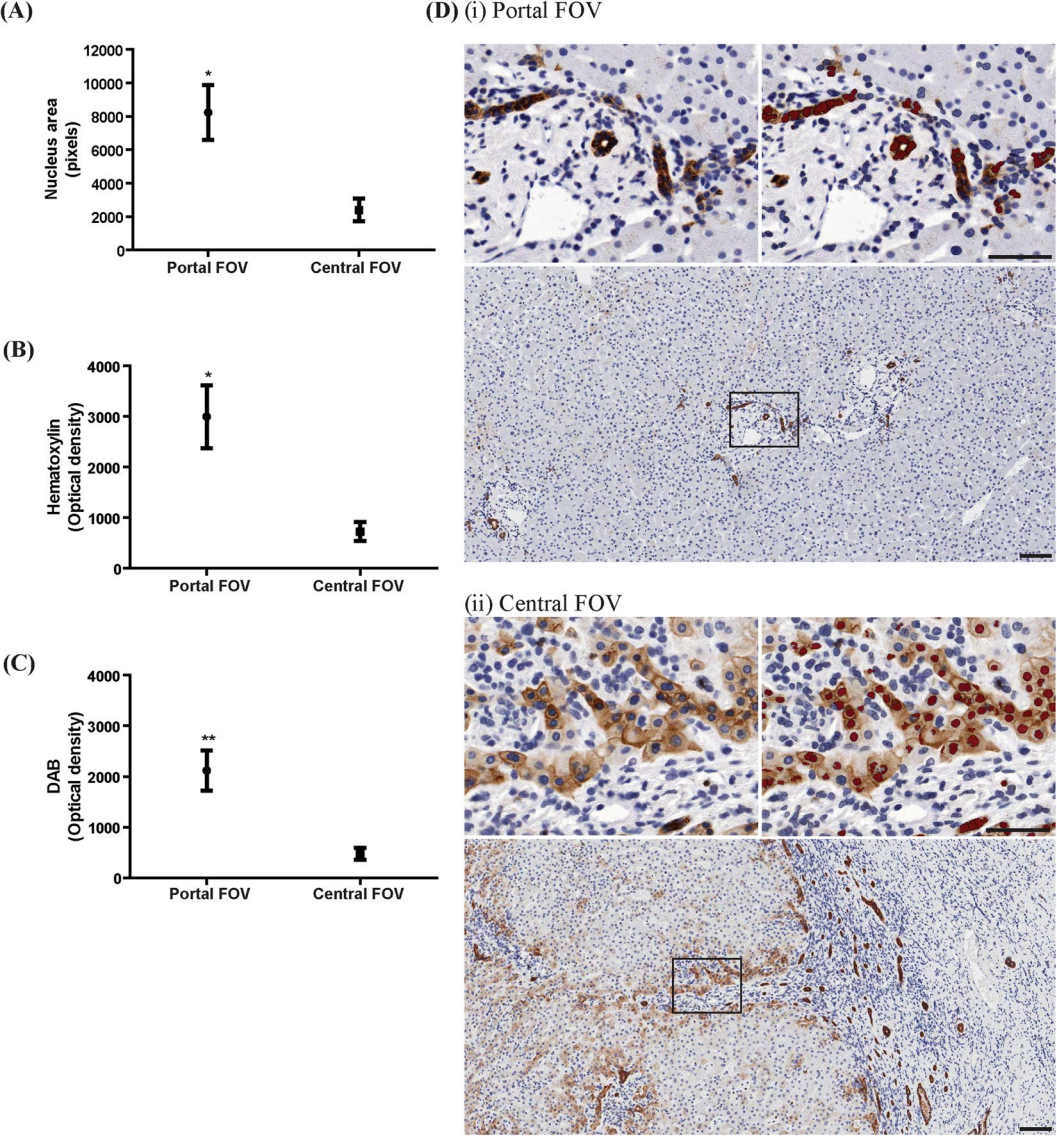
The custom-designed PCK algorithm can determine whether an entire FOV predominantly consists of ductal or non-ductal PCK^+ve^ cells. (**A**–**C**) Portal FOVs have significantly higher nucleus area, and higher optical density of hematoxylin and DAB. (**D**) Representative images of portal and central FOVs, with red nuclei indicating PCK^+ve^ cells counted by InForm. n = 16 FOVs per group. Scale bar 100 μm. *p < 0.05, **p < 0.001.

Custom algorithms can be configured to identify and quantify presumptive HPCs stained with two labelling techniques; immunohistochemistry and immunofluorescence. For immunohistochemistry, three normal livers and four livers from the hepatitis cohort were stained for Pan Cytokeratin (PCK); a general epithelial stain that cross reacts with a wide range of cytokeratins, which have been used as a marker for cholangiocytes and HPCs^35–37^. InForm was configured to distinguish PCK^+ve^ cells by following PerkinElmer’s workflow to create and verify an algorithm (“PCK phenotype IHC algorithm”; see Supplementary Methods Table S1 and Table S2) on fifteen field of views (FOVs) of both normal and hepatitis liver. The algorithm correctly identified all PCK^+ve^ cells, and distinguishes these from PCK^−ve^ cells based on optical density of DAB (p < 0.0001, Fig. 1A). Additionally, we sought to verify the accuracy of InForm by comparing it to manual counting. Fifteen FOVs were first processed with InForm, then counted manually by a single blinded investigator. We report a high correlation of InForm’s automated output with manual counting (r^2^ = 0.9202, p < 0.0001, Fig. 1C). Next, we examined whether the algorithm could distinguish ductal PCK^+ve^ cells from non-ductal PCK^+ve^ cells. Using Adobe Photoshop, we extracted individual PCK^+ve^ ductal and PCK^−ve^ non-ductal cells from the images, analysed these in InForm using the previously created PCK algorithm. Ductal cells could be distinguished from non-ductal cells based on parameters including nuclear areas and the optical density of hematoxylin and DAB staining (p < 0.0001, Fig. 2). In addition to single-cell analysis, we tested whether the algorithms could distinguish ductal PCK^+ve^ cells and non-ductal PCK^+ve^ presumptive HPCs in whole FOVs. Sixteen portal FOVs (consisting of predominantly PCK^+ve^ ductal cells) and sixteen central FOVs (populated mainly by non-ductal PCK^+ve^ cells) were analysed with InForm. This confirmed that InForm outputs consistently assigned ductal cells to portal areas, and non-ductal cells to central regions of the FOVs (p < 0.05, Fig. 3A,B and p < 0.001, Fig. 3C).

**Figure 4:**
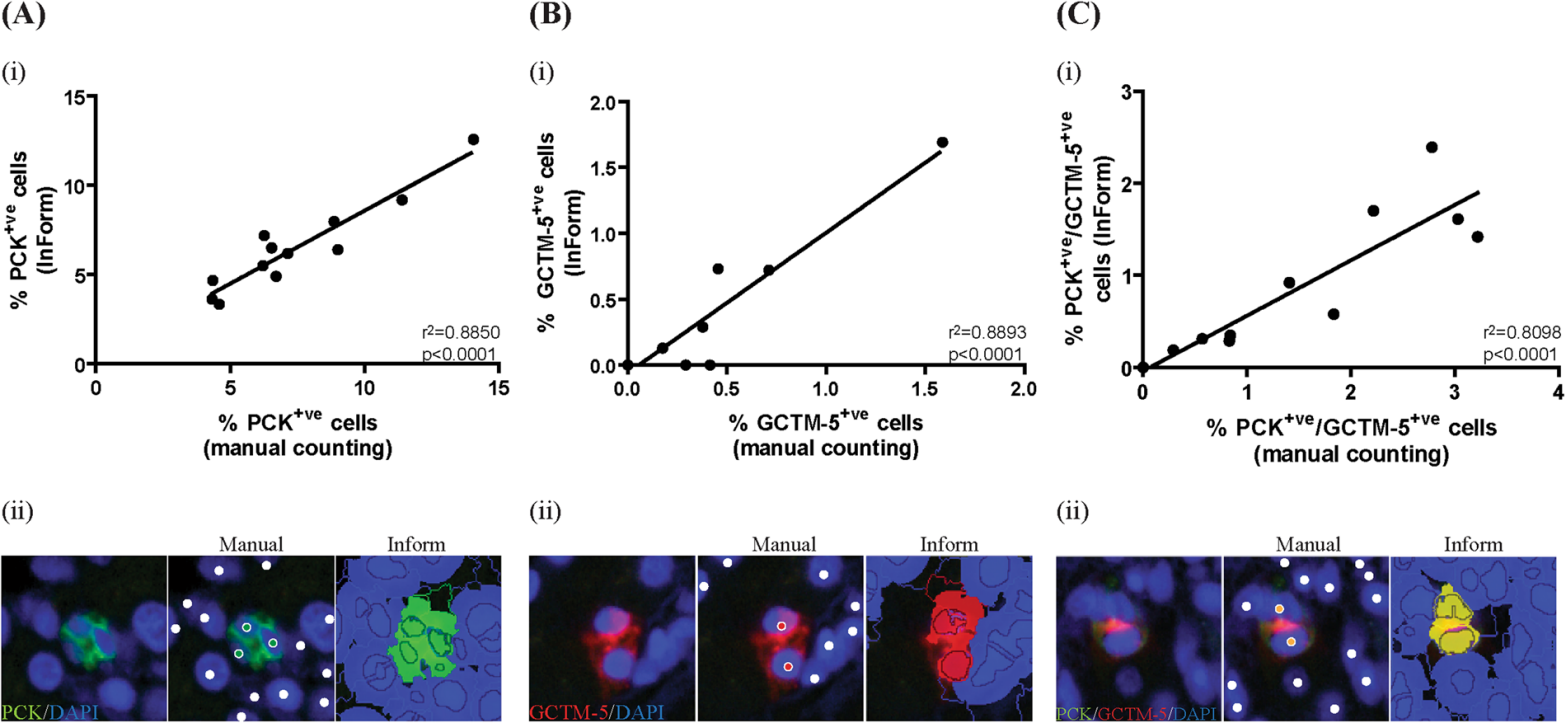
A custom algorithm was created to quantitate the number of PCK^+ve^ and GCTM-5^+ve^ cells stained using double immunofluorescence labelling. (**Ai**–**Bi**) InForm outputs had a high correlation compared to manual counting in both channels (r^2^ = 0.8850 and r^2^ = 0.8893, p < 0.0001. (**Ci**) Double positive cells also have a high correlation with manual counting (r^2^ = 0.8098, p < 0.0001). (**Aii**–**Cii**) Comparative images of cells counted manually and with InForm are shown. For manual counts, green dots indicate PCK^+ve^ cells, red dots indicate GCTM-5^+ve^ cells, yellow dots indicate PCK^+ve^/GCTM-5^+ve^ cells and white dots indicate nuclei of cells that negative. For InForm counts, PCK^+ve^, GCTM-5^+ve^, double positive cells and double negative are shown in green, red, yellow and blue respectively. n = 12 FOVs.

Next, we evaluated whether InForm can be configured to accurately identify fluorescently double-labelled presumptive HPCs. Three normal and three hepatitis livers were stained for two HPC markers (PCK and GCTM-5). Two FOVs per sample were scored both manually and with InForm software. A high correlation with manual counting was reported in both channels, (green: r^2^ = 0.8850; red: r^2^ = 0.8893, p < 0.0001, Fig. 4A,B), and with double positive cells (r^2^ = 0.8098, p < 0.0001, Fig. 4C).

### Custom algorithms can identify histological features of pathological significance

**Figure 5:**
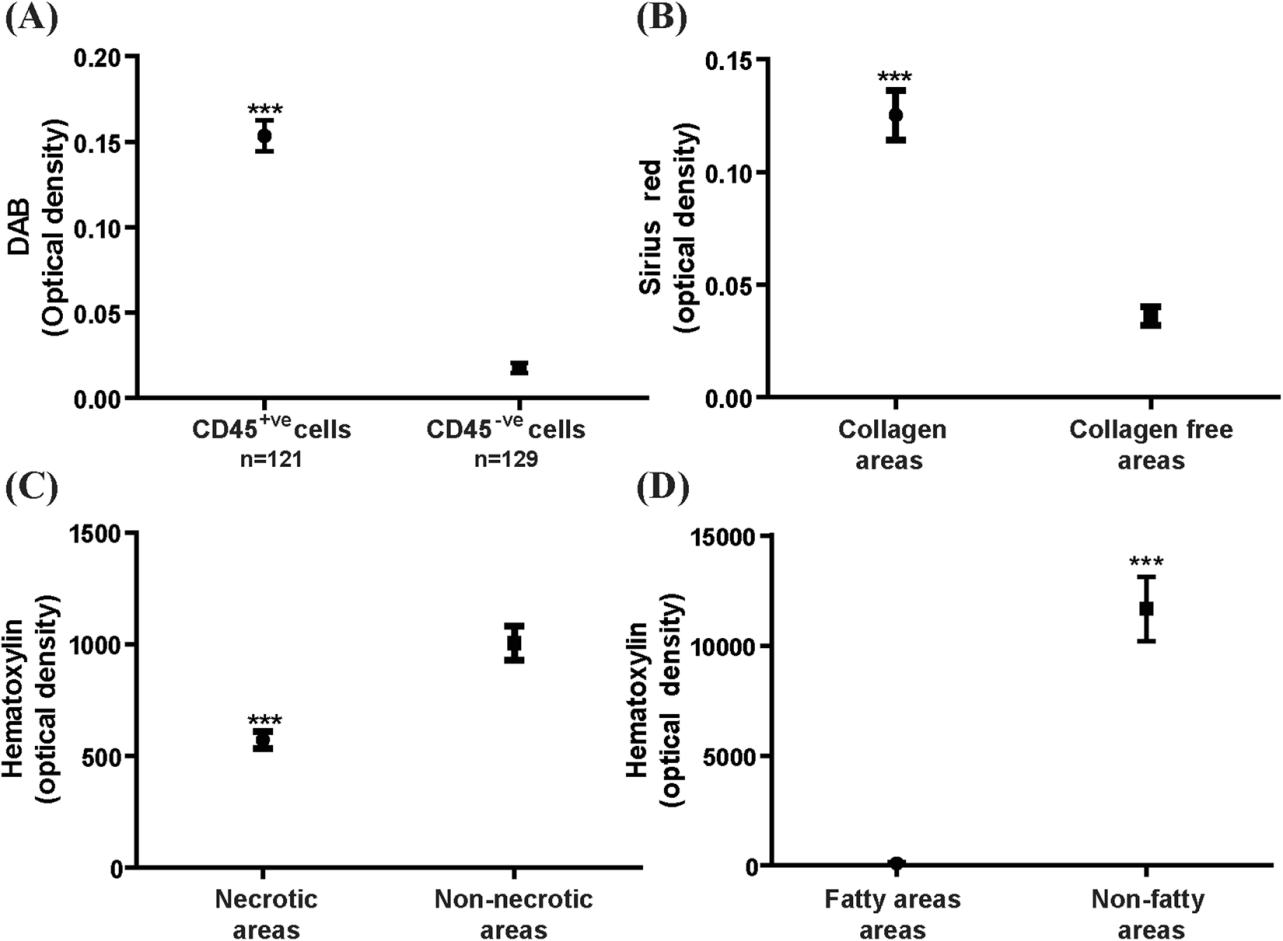
Custom algorithms were created to identify histological features of pathological significance. (**A**) CD45^+ve^ inflammatory cells have higher optical density of DAB compared to CD45^−ve^ cells. (**B**) Areas with collagen deposition have higher optical density of Sirius Red compared to collagen free areas. (**C**,**D**) Necrotic areas and fatty areas have lower optical density of hematoxylin compared to parenchyma lacking these features. For (**B**–**D**), ten FOVs from each sample was used for analysing fibrotic, fatty and necrotic areas, and compared to ten FOVs of parenchyma lacking these features. ***p < 0.0001.

**Figure 6:**
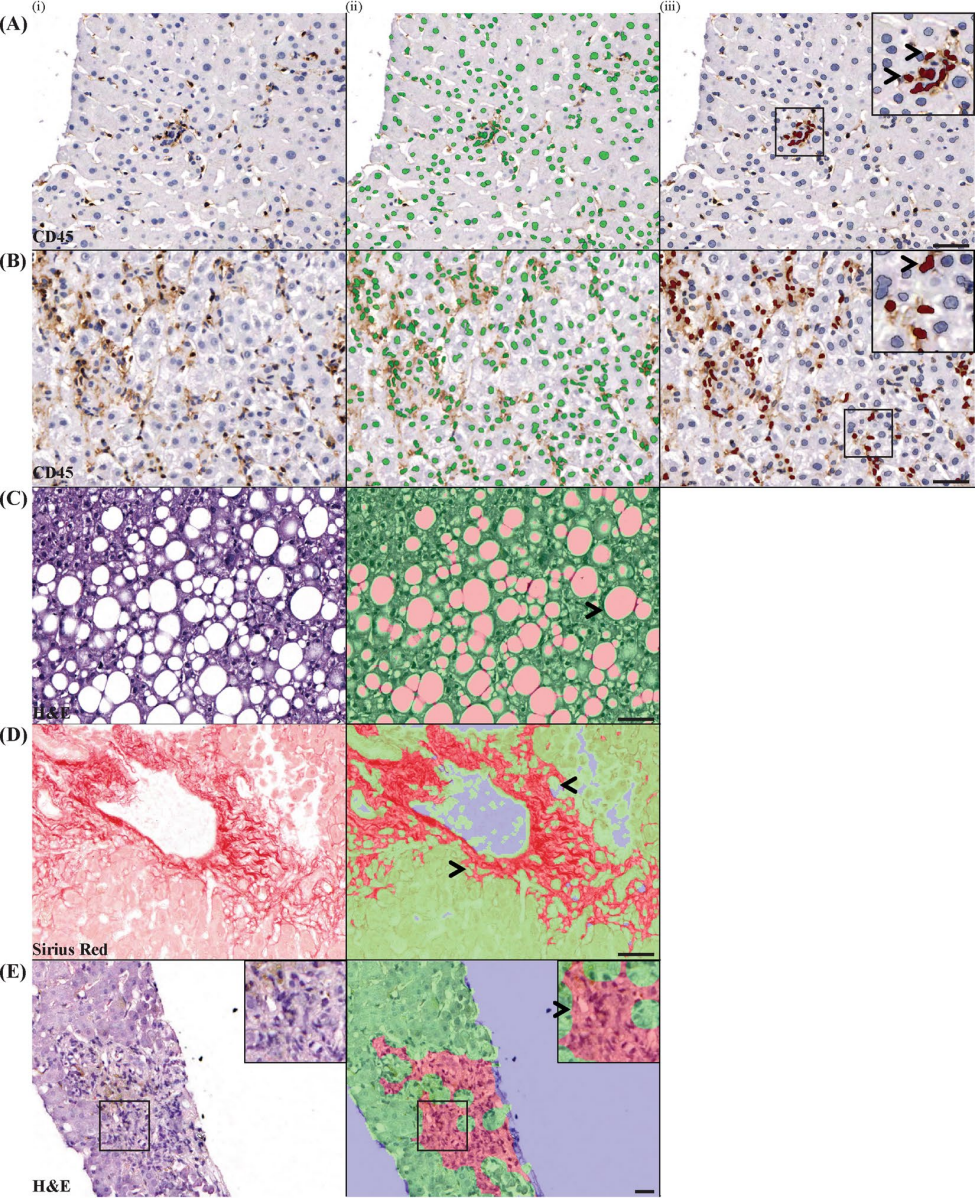
Custom algorithms can identify histological features of pathological significance. Both inflammatory foci (**A**) and individual inflammatory cells (**B**) can be identified using InForm, which is necessary in order to score piecemeal, lobular and portal inflammation on the Ishak-Knodell scale. Areas of fat (**C**), collagen deposition (**D**) and areas of confluent necrosis (**E**) can also be identified using InForm algorithms. The original images are shown in (i), and the InForm mark-ups are shown in (ii) and (iii). Green indicates cells/areas that are negative for feature of interest, and pink/red indicates positive cells/areas. Scale bar 100 μm.

InForm can also be configured to identify important histological features that assist in the grading of liver disease. The acute hepatitis samples were stained in series with CD45 and H&E to identify inflammatory cells and areas of necrosis respectively. The NAFLD samples were stained with CD45, Sirius Red and H&E to identify inflammatory cells, collagen deposition and areas of fat respectively. Three normal livers were stained and used as controls. Histological features were identified and marked by the pathologist (B.L). For each feature at least 10 different areas were used for the creation of each algorithm. Note that each histological feature required the creation and verification of a separate algorithm (see Supplementary Methods Tables S1 and S2). Using these algorithms, we consistently identified CD45^+ve^ inflammatory cells, and areas of collagen deposition, fat and necrosis, based on the optical density of DAB, Sirius Red and haematoxylin respectively (p < 0.0001, Figs 5 and 6).

### InForm outputs correlate with Pathologist’s scoring of necroinflammatory activity

**Figure 7:**
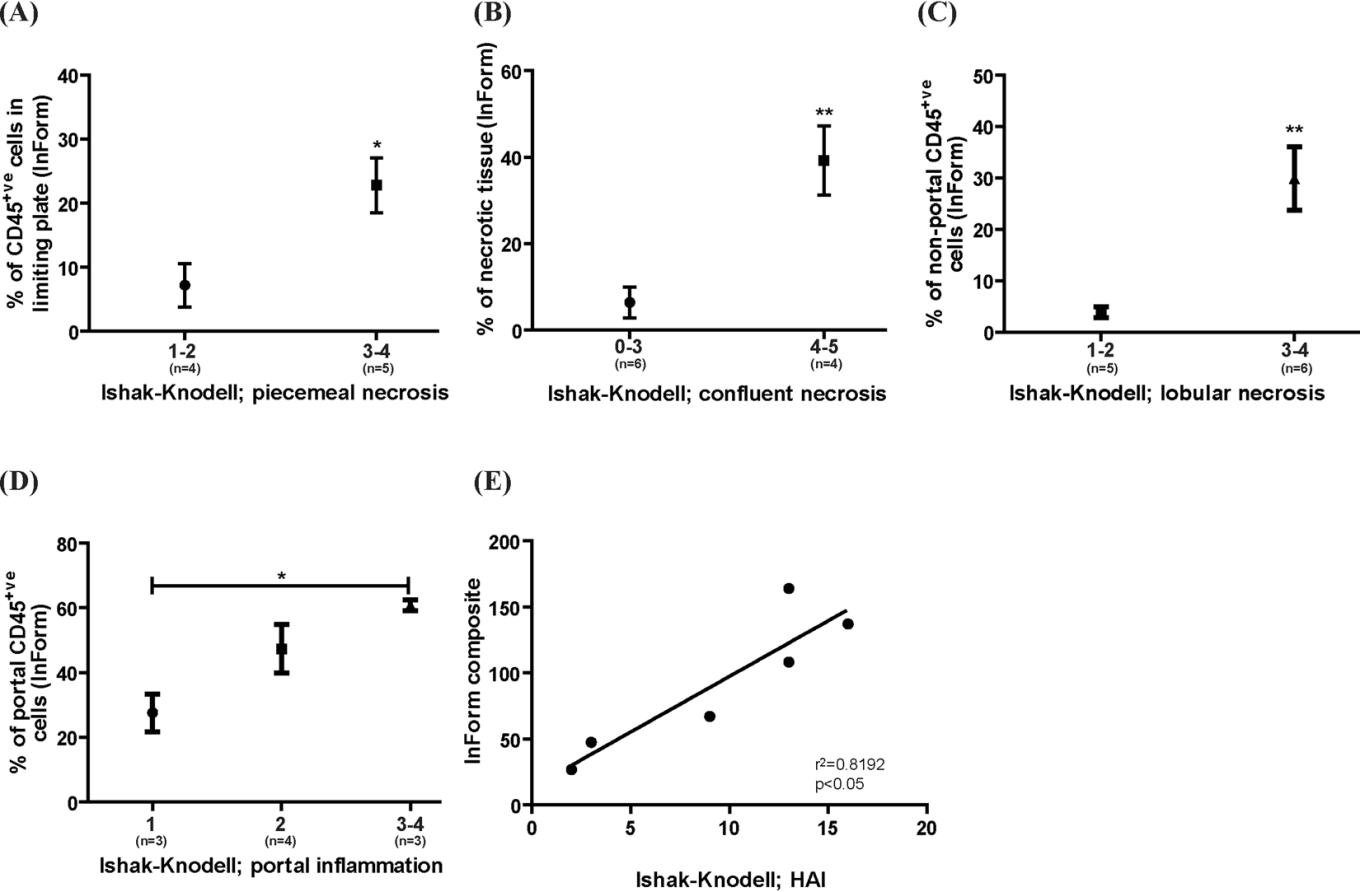
InForm outputs correlate with Ishak-Knodell scores assessing necroinflammatory activity. (**A**–**D**) For all categories, a higher Ishak-Knodell score (indicative of a more severe pathology) resulted in a significantly higher InForm score. (**E**) To obtain the InForm composite, values from all categories were added. A high correlation between the HAI and the InForm composite score is evident (r^2^ = 0.8192, p < 0.05). *p < 0.05, **p < 0.001.

We next investigated whether InForm’s output correlated with a pathologist’s assessment of necroinflammatory activity. Eleven acute hepatitis samples were scored with the Ishak-Knodell system by a Pathologist (B.L.) (see Supplementary Methods Table S3). To assess each category of the Ishak-Knodell system using InForm, we applied the algorithms created previously (see Supplementary Methods Table S1 for parameters) in combination with processing regions to create a surrogate measure. These surrogate measures were necessary as the Ishak-Knodell system provide a descriptive evaluation of the liver mainly in architectural changes (Supplementary Methods Table S3). An explanation of the surrogate measures is given in Supplementary Methods Table S4. For all categories, a higher Ishak-Knodell score (reflecting a more severe pathology) resulted in a significantly higher InForm score (p < 0.05, Fig. 7A,D, and p < 0.001, Fig. 7B,C). To obtain the InForm composite score, values from all categories were added, similar to the HAI. A high correlation between the HAI and the InForm composite score was found (r^2^ = 0.8192, p < 0.05), demonstrating that InForm can be used to generate a score that represents the severity of necroinflammatory activity (Fig. 7E).

## Discussion

This study establishes InForm as a useful research tool for assessing liver pathology. We demonstrate that custom algorithms can be designed and used to quantitate ductal PCK^+ve^ cells and non-ductal PCK^+ve^ presumptive HPCs, CD45^+ve^ cells, as well as identify areas of fat, necrosis, and collagen deposition. It can produce an objective score comparable to Ishak-Knodell’s HAI to represent liver inflammation severity.

InForm can reliably quantitate presumptive HPCs with a high correlation to manual counting using either immunohistochemically or immunofluorescently stained samples. Importantly, InForm can also be configured to objectively distinguish ductal cells staining positive for HPC markers from centrally located non-ductal presumptive HPCs; a useful tool for researchers who wish to independently assess the ductal reaction from the HPC response^38^.

The software’s semi-automated batch workflow allowed for rapid and objective processing of samples, in contrast to the time-consuming and subjective manual counting of HPCs. Many studies in the literature ascertain the HPC response by counting HPCs manually^11,23,38–42^. We have shown in this study that this process can be reliably automated using InForm, to allow for rapid and objective processing of samples. To circumvent manual counting, researchers have also utilised various software programs to calculate the area occupied by the cells of interest, expressed as a percentage of total tissue^37,42^. Area calculations on stained tissue may be confounded by several factors including tissue distortions due to sinusoidal enlargement, fixation, or surgical artefacts^43^. In contrast, InForm offers a more reliable output (number of positive cells expressed as a percentage of total cells), for which we report a higher correlation to manual counting compared to area calculations using Aperio’s ImageScope software (see Supplementary Results Fig. S1). It is likely that InForm can identify and quantify any given cell of interest in any species, providing an antibody exists to mark it, and/or it has a distinct appearance in a histological stain. For instance, we also applied InForm algorithms to mouse studies to accurately quantitate the numbers of PCK^+ve^ presumptive HPCs, F4/80^+ve^ macrophages and CD45^+ve^ inflammatory cells (see Supplementary Results Fig. S2). Hence, InForm can also be used to study rodent models of HCC.

InForm can also be configured to score inflammatory activity. Algorithms can be customised to score the number of inflammatory cells (either single cells or aggregates), within portal or lobular regions or within the limiting plates to produce outputs that correlate with Ishak-Knodell scores for portal, lobular or piecemeal necrosis respectively. However, we were unable to create an algorithm that recognises inflammatory cells in H&E stained samples; CD45 staining was necessary, although conceivably any other stain for inflammatory cells would suffice. Areas of necrosis can also be assessed using customised algorithms, with the output correlating with Ishak-Knodell scores for confluent necrosis. Importantly, algorithms can be archived and shared among researchers, promoting reproducibility and efficiency.

In summary, the InForm software package offers an effective tool for liver researchers studying HPCs in the context of inflammation, fibrosis and fat. We propose that InForm can replace not only manual counting of HPCs, but also the manual assessment of liver disease in a plethora of studies involving a range of etiologies and models^11,12,38,44^. The major advantages of InForm include objectivity, reproducibility, efficiency and high throughput, and it provides a solution to the economical and logistical challenges of involving pathologists in large-scale research studies. Using InForm, researchers can produce a single composite score to represent necroinflammatory severity, thus providing a useful tool for liver disease research.

## Methods

### Patient cohort

This study was approved by the ethics committees of the University College of London, University of Queensland, Sir Charles Gairdner Hospital, Metro South Hospital, and the University of Western Australia. All methods were carried out in accordance to the guidelines and regulations of these ethics committees. Informed consent was obtained from patients when required under the relevant ethics regulation, and in other cases this was waived by the relevant ethics committee. Formalin-fixed, paraffin-embedded archival liver samples were obtained from University College of London, University of Queensland, and Sir Charles Gairdner Hospital. We examined cohorts of acute hepatitis (n = 11, classified as either viral, drug induced or autoimmune acute hepatitis), non-alcoholic fatty liver disease (NAFLD; n = 3) and normal livers (n = 5) as controls. See Supplementary Methods Table S6 for cohort information.

### Immunohistochemistry

Serial sections of liver biopsies (4 μm thick) were mounted on positively charged microscope slides. Sections were dewaxed, rehydrated, and submerged into pre-heated (98 °C) antigen-retrieval citrate buffer (10 mM, pH6.0) for 30 min, followed by a further 20 min incubation at room temperature. Endogenous peroxidase activity was quenched by treating with 3% H_2_0_2_ for 10 min, followed by the application of DAKO’s biotin blocking system (DAKO, Cat.X0590) and then serum-free protein block (Dako, Cat.X0909) as per the manufacturer’s instruction. The primary antibody was applied overnight at 4 °C (PCK; DAKO, Cat.Z0622, 1:800 dilution or CD45; DAKO, Cat.M0701, 1:50 dilution). Note that primary antibodies were diluted in antibody diluent (Dako, Cat S2022). Staining was detected with the LSAB+ kit (Dako, Cat.K0690) and visualized with DAB + substrate (Dako, Cat.K0690) as per manufacturer’s instructions. Slides were counterstained with Harris’ hematoxylin, dehydrated and mounted using DPX mounting medium. Positive controls (normal livers with bile ducts) and negative controls for antigen, primary and secondary antibodies were included for all immunohistochemical experiments.

### Double labelling indirect immunofluorescence

Samples were dewaxed, rehydrated, antigen retrieved and blocked as per immunohistochemical protocol. The primary antibodies were applied overnight at 4 °C (PCK; DAKO, Cat.Z0622, 1:600 dilution and GCTM-5; Millipore Cat.MAB4365, 1:100 dilution). Staining was detected with Alexa Fluor® dyes goat anti rabbit AF488 and goat anti mouse AF594 (Life Technologies, Cat.A11005 and Cat.A11008, 1:400). Note that all antibodies were diluted in DAKO’s antibody diluent (Dako, Cat S2022). Samples were counterstained with DAPI and sealed by a coverslip using an aqueous mounting media (Gelvatol). Positive controls (normal livers with bile ducts) and negative controls for antigen, primary and secondary antibodies were included for all immunofluorescence experiments.

### Histological stains

Samples were dewaxed and rehydrated as per immunohistochemical protocol described above. For H&E, a regressive hematoxylin (Harris’ modified hematoxylin, Sigma, Cat.HHS128) and Eosin Y (Sigma, Cat.230251) protocol was performed. In brief, slides were submerged in hematoxylin for 80 sec, followed by a 3 sec submersion into 1% acid alcohol solution, and 2 min incubation in Scott’s tap water (pH8.0). For Sirius Red, samples were submerged in Picro’s Sirius Red (Abcam, Cat.ab150681) for 1 h, followed by a 1 min 0.1 N HCl wash. Slides were dehydrated and mounted using DPX.

### Image acquisition & processing

All samples were scanned at 20x-magnification using the Aperio Scanscope XT. ImageScope (v12.0.0.5) was used to view images and extract at least 15 FOVs (at 20x-magnification) per sample, depending on the size of the biopsy. Extracts were saved as TIFF files. For verification of algorithms, images were pre-processed with Adobe Photoshop (CS3, V10.0) to outline and crop the cell/area of interest, then exported as TIFF files and imported into InForm (v2.0.4743.16069). For all other applications, TIFF files were directly imported into InForm for analysis. Algorithms were created according to parameters shown in Supplementary Methods Table S1, and verified (see Supplementary Methods Table S2 for workflow). Data was exported as.txt files and imported into Microsoft Excel. For pixel counting using Aperio ImageScope, see Supplementary Methods and Supplementary Methods Table S5.

### Manual counting and scoring using Ishak-Knodell

For manual counting of PCK^+ve^ and GCTM-5^+ve^ HPCs, TIFF images were exported into ImageJ (v1.51 h, Java1.8.0_66). The cell counter plugin was used to keep track of counted cells and the data was exported into Excel. All manual counting was performed blind, by a single investigator for consistency purposes. For scoring of the liver biopsies, H&E stained samples were provided for assessment to Pathologist B.L., whom identified areas of fat, fibrosis and inflammation and scored samples using the Ishak-Knodell system (see Supplementary Methods Table S3).

### Data availability

The datasets analysed during the current study are available from the corresponding author on reasonable request.

## Electronic supplementary material

Supplementary Information
