# Supplementary material for: InForm software: a semi-automated research tool to identify presumptive human hepatic progenitor cells, and other histological features of pathological significance
Source: Sci Rep. 2018 Feb 21;8:3418. doi: 10.1038/s41598-018-21757-4 (PMC5821869; doi:10.1038/s41598-018-21757-4)
Supplement: Supplementary file 1 — Supplementary Information [file 41598_2018_21757_MOESM1_ESM.doc]

**InForm software: a semi-automated research tool to identify presumptive human hepatic progenitor cells, and other histological features of pathological significance**

Anne S. Kramer^1,2,3^, Bruce Latham^4^, Luke A. Diepeveen^1^, Lingjun Mou^5^, Geoffrey J Laurent^3^, Caryn Elsegood^6^, Laura Ochoa-Callejero^7^ and George C. Yeoh^1, 2,3^,*

^1^Harry Perkins Institute of Medical Research, QEII Medical Centre, Nedlands and Centre for Medical Research, The University of Western Australia, Crawley, WA

^2^School of Molecular Sciences, University of Western Australia, Crawley, WA

^3^Centre for Cell Therapy and Regenerative Medicine, School of Biomedical Science, The University of Western Australia, Crawley, WA

^4^PathWest Laboratory Medicine WA, Fiona Stanley Hospital, Murdoch, WA

^5^WA Liver & Kidney Surgical Transplant Service, Sir Charles Gairdner Hospital

^6^ School of Biomedical Science, Curtin Health Innovation Research Institute, Curtin University, Bentley, WA

^7^Angiogenesis group, Oncology Area, Centre for Biomedical Research of La Rioja, Logroño, Spain

**Corresponding author*

**Supplementary Information**

**Supplementary Results**


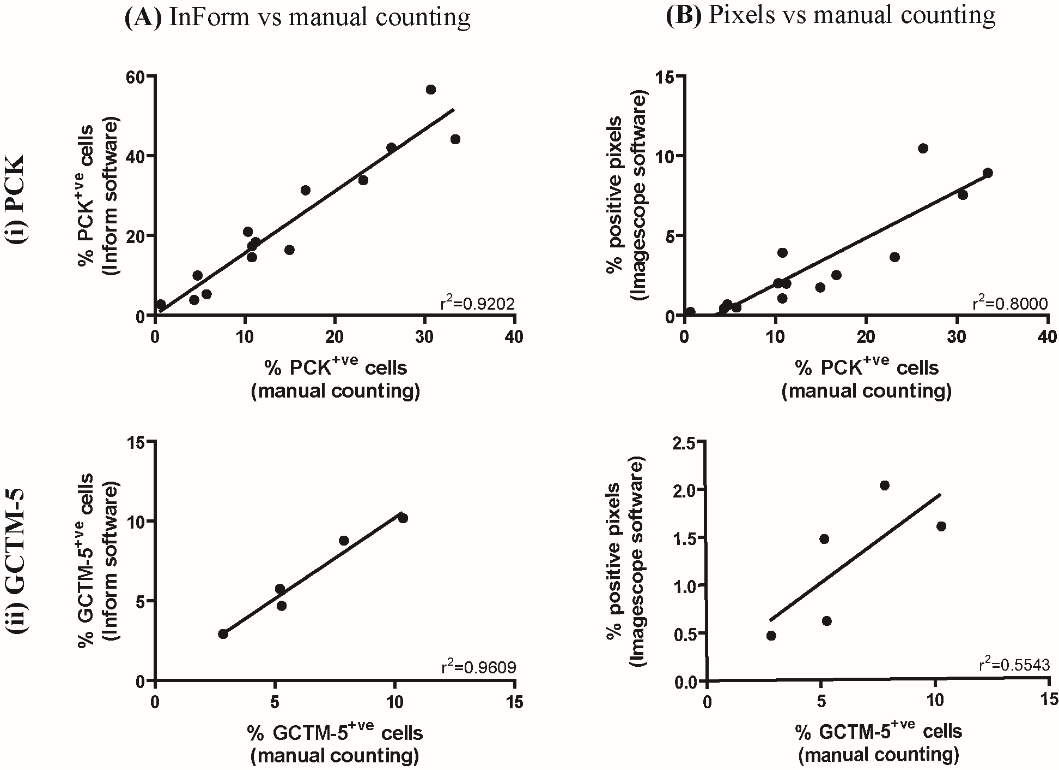


**Supplementary Results Figure S1:** A comparison of manual counting to two automated methods; PerkinElmer’s InForm software package (v2.0.4743.16069) to calculate the percentage of positive cells and Aperio ImageScope software (V12.0) to calculate the percentage of positive pixels. (A) InForm was used to calculate the number of presumptive HPCs as a percentage of total cells, and we report a high correlation to manual counting with both PCK (r^2^=0.9202) (Ai) and GCTM-5 (r^2^=0.9609) (Aii). Pixel counting using ImageScope software has a high correlation for PCK (r^2^=0.8000) (Bi) but not with GCTM-5 (r^2^=0.5543) (Bii). This suggests that area output is not as accurate compared to counting the number of positive cells as a percentage of total cells.


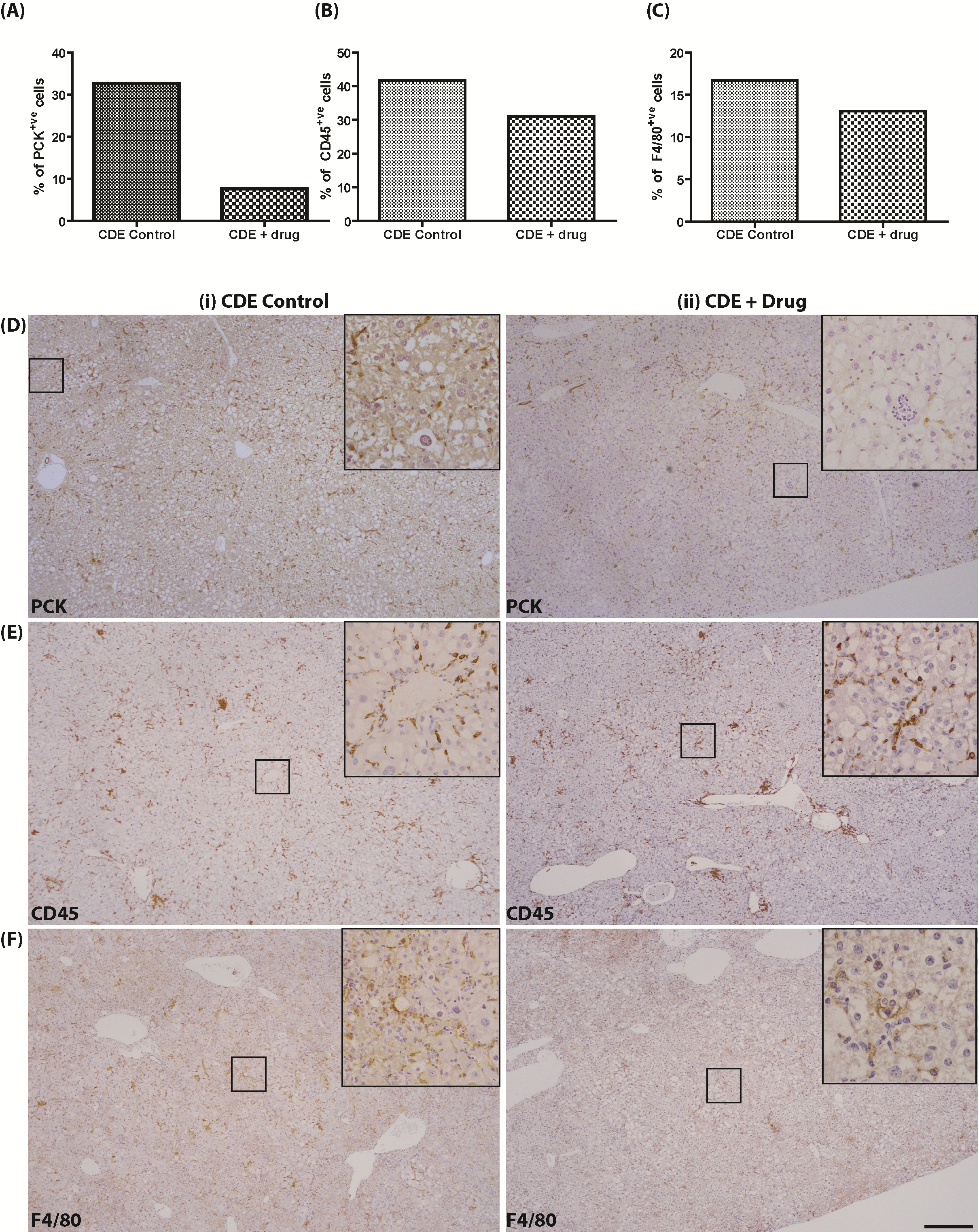


**Supplementary Results Figure S2**. InForm can be utilised to examine HPCs and the inflammatory response in mouse studies. Mice were placed on a long-term choline-deficient, ethionine supplemented (CDE) diet to induce chronic liver damage, and stimulate HPC-mediated repair. Mice were treated with a drug and the HPC and immune response was assessed by PCK, CD45 and F4/80 immunohistochemical staining and analysed using InForm algorithms (A-C). Preliminary data shows a trend for a reduction of the HPC and immune response in mice with CDE + drug, compared to CDE diet alone. Representative images of CDE control (i) and CDE + drug of PCK, CD45 and F4/80 are shown in D-F. Scale bar 200μm.

**Supplementary Methods**

**Supplementary Methods Table S1**: Algorithm parameters.

| **Name of algorithm** | **Setting Category** | **Settings** |
| --- | --- | --- |
| **PCK phenotype IHC** | Images analysed | PCK stained sections (IHC) |
|  | Configuration settings | Segment images>trainable tissue segmentation  Find features>cell segmentation  Score>Score |
|  | Image preparation settings | Image format>RGB  Sample resolution>Brightfield  Convert to optical density>select white  Spectral library>Brightfield  Spectra for unmixing>blue hematox, DAB |
|  | Tissue segmentation settings | Tissue categories>PCK, parenchyma, white  Components for training>blue hematox, DAB  Pattern scale>small  Train tissue segmenter>99%  Segmentation resolution>fine  Trim edges>3pixels, PCK category |
|  | Cell segmentation settings | Compartments to segment>nuclei  Edge rules>discard if touching edge  Nuclei segmentation  >Tissue category>all categories  >approach>object based  >signal scaling>auto scale  >Primary>blue hematox>min signal 0.25  >size range  >min size 150 pixels  >max sixe 10000 pixels  >clean-up  >fill holes  >refine splitting  >roundness min circularity 0.3 |
|  | Scoring setting | Tissue category>PCK  Scoring>Positivity (2-bin)  Compartment>Nuclei  Component>DAB  Threshold max>0.4  Positivity threshold>0 |
| **PCK/GCTM-5 fluorescence** | Images analysed | PCK/GCTM-5 stained sections (fluorescence)  PCK – green channel  GCTM-5 – red channel |
|  | Configuration settings | Segment images>skip  Find features>cell segmentation  Score>Score |
|  | Image preparation settings | Image format>RGB  Sample resolution>Fluorescence  Spectral library>Fluorescence  Spectra for unmixing>red, green, blue  Scaling for unmixed data counts>raw |
|  | Cell segmentation settings | Compartments to segment>nuclei, cytoplasm, membrane  Nuclei segmentation  >approach>object based  >signal scaling>auto scale  >Primary>blue >min signal 0.23  >size range  >min size 20 pixels  >max sixe 10000 pixels  >clean-up  >fill holes>max hole 50 pixels  >refine splitting  >roundness min circularity 0.15  Cytoplasm segmentation  >Inner distance to nucleus>0 pixels  >outer distance to nucleus>15 pixels  >Minimum size>1 pixel  Membrane segmentation  >primary>green>full scale counts 61.97  >secondary>red>full scale counts 72.50  >segmentation priority>assign to membrane  >maximum cell size>distance to membrane>40 pixels |
|  | Scoring setting | Scoring>Double Positivity (2x2-bin)  First marker setting (GCTM-5)  >Compartment>Membrane  >Component>red  >Threshold max>80  >Positivity threshold>18.4  Second marker setting (PCK)  >compartment>membrane  >component>green  >threshold max>12  >positivity threshold>7 |
| **CD45 piecemeal necrosis** | Images analysed | CD45 stained sections (IHC) |
|  | Configuration settings | Segment images>trainable tissue segmentation  Find features>cell segmentation  Score>Score |
|  | Image preparation settings | Image format>RGB  Sample resolution>Brightfield  Convert to optical density>select white  Spectral library>Brightfield  Spectra for unmixing>blue hematox, DAB |
|  | Tissue segmentation settings | Tissue categories>CD45, parenchyma, white  Components for training>blue hematox, DAB  Pattern scale>medium  Train tissue segmenter>94%  Segmentation resolution>fine  Trim edges>5pixels, CD45 category |
|  | Cell segmentation settings | Compartments to segment>nuclei  Nuclei segmentation  >Tissue category>all categories  >approach>object based  >signal scaling>auto scale  >Primary>blue hematox>min signal 0.18  >size range  >min size 80 pixels  >max sixe 10000 pixels  >clean-up  >fill holes  >refine splitting  >roundness min circularity 0.25 |
|  | Scoring setting | Tissue category>CD45  Scoring>Positivity (2-bin)  Compartment>Nuclei  Component>DAB  Threshold max>0.5  Positivity threshold>0.063 |
|  | Processing regions | Set to 60-70μm margin around portal tracts. |
| **CD45 lobular necrosis** | Images analysed | CD45 stained sections (IHC) |
|  | Configuration settings | Segment images>trainable tissue segmentation  Find features>cell segmentation  Score>Score |
|  | Image preparation settings | Image format>RGB  Sample resolution>Brightfield  Convert to optical density>select white  Spectral library>Brightfield  Spectra for unmixing>blue hematox, DAB |
|  | Tissue segmentation settings | Tissue categories>CD45 foci, parenchyma, white  Components for training>blue hematox, DAB  Pattern scale>medium  Train tissue segmenter>93%  Segmentation resolution>medium  Trim edges>10pixels, CD45 foci category  Min segment size>1200 pixels |
|  | Cell segmentation settings | Compartments to segment>nuclei  Edge rules>discard if touching edge  Nuclei segmentation  >Tissue category>all categories  >approach>object based  >signal scaling>auto scale  >Primary>blue hematox>min signal 0.15  >size range  >min size 100 pixels  >max sixe 500 pixels  >clean-up  >fill holes  >refine splitting  >roundness min circularity 0.25 |
|  | Scoring setting | Tissue category>CD45 foci  Scoring>Positivity (2-bin)  Compartment>Nuclei  Component>DAB  Threshold max>0.25  Positivity threshold>0 |
|  | Processing regions | Set to exclude portal areas |
| **CD45 portal inflammation** | Images analysed | CD45 stained sections (IHC) |
|  | Configuration settings | Segment images>trainable tissue segmentation  Find features>cell segmentation  Score>Score |
|  | Image preparation settings | Image format>RGB  Sample resolution>Brightfield  Convert to optical density>select white  Spectral library>Brightfield  Spectra for unmixing>blue hematox, DAB |
|  | Tissue segmentation settings | Tissue categories>CD45, parenchyma, white  Components for training>blue hematox, DAB  Pattern scale>medium  Train tissue segmenter>94%  Segmentation resolution>fine  Trim edges>2pixels, CD45 category |
|  | Cell segmentation settings | Compartments to segment>nuclei  Nuclei segmentation  >Tissue category>all categories  >approach>object based  >signal scaling>auto scale  >Primary>blue hematox>min signal 0.18  >size range  >min size 80 pixels  >max sixe 10000 pixels  >clean-up  >fill holes  >refine splitting  >roundness min circularity 0.25 |
|  | Scoring setting | Tissue category>CD45 foci  Scoring>Positivity (2-bin)  Compartment>Nuclei  Component>DAB  Threshold max>0.5  Positivity threshold>0.063 |
|  | Processing regions | Set around portal triads |
| **H&E confluent necrosis** | Images analysed | H&E stained sections |
|  | Configuration settings | Segment images>trainable tissue segmentation  Find features>cell segmentation  Score>Skip |
|  | Image preparation settings | Image format>RGB  Sample resolution>Brightfield  Convert to optical density>select white  Spectral library>Brightfield  Spectra for unmixing>blue hematox, eosin |
|  | Tissue segmentation settings | Tissue categories>confluent necrosis, parenchyma, white  Components for training>blue hematox, eosin  Pattern scale>medium  Train tissue segmenter>87%  Segmentation resolution>fine  Trim edges>10pixels, confluent necrosis category  Min segment size>500 pixels |
|  | Cell segmentation settings | Compartments to segment>nuclei  Nuclei segmentation  >Tissue category>all categories  >approach>object based  >signal scaling>auto scale  >Primary>blue hematox>min signal 0.25  >size range  >min size 80 pixels  >max sixe 1500 pixels  >clean-up  >fill holes  >refine splitting  >roundness min circularity 0.28 |
|  | Processing regions | Set to exclude portal regions |
| **Sirius Red** | Images analysed | Sirius red stained sections |
|  | Configuration settings | Segment images>trainable tissue segmentation  Find features>skip  Score>Skip |
|  | Image preparation settings | Image format>RGB  Sample resolution>Brightfield  Convert to optical density>select white  Spectra for unmixing>blue, green, red |
|  | Tissue segmentation settings | Tissue categories>Sirius Red, parenchyma, white  Pattern scale>small  Train tissue segmenter>99.2%  Segmentation resolution>extra fine  Trim edges>4pixels, Sirius red category  Min segment size>500 pixels |
| **H&E steatosis** | Images analysed | H&E stained sections |
|  | Configuration settings | Segment images>trainable tissue segmentation  Find features>skip  Score>Skip |
|  | Image preparation settings | Image format>RGB  Sample resolution>Brightfield  Convert to optical density>select white  Spectra for unmixing>blue hematox, eosin |
|  | Tissue segmentation settings | Tissue categories>macro steatosis, parenchyma  Pattern scale>medium  Train tissue segmenter>90%  Segmentation resolution>extra fine  Trim edges>10pixels, macro steatosis category |
|  | Processing regions | Set to exclude white space |

**Supplementary Methods Table S2**: Workflow of algorithm creation and verification

| **Process** | **Steps** |
| --- | --- |
| **Create Algorithm** | 1. Load at least 10 images that includes a representative range.  - Includes samples from all groups - Includes samples that show high and low amount of feature of interest - Includes samples that have low and high background levels  1. Set parameters (given in Supplementary Methods Table S1) 2. Set training regions and processing regions (given in Supplementary Methods Table S1) 3. Train tissue segmenter. Ensure accuracy rate is above 80%. 4. Save algorithm |
| **Verify Algorithm** | 1. In Adobe Photoshop, outline and crop individual cells/areas that have the feature of interest (e.g. clusters of inflammatory cells) and also outline and crop cells/areas that do not show this feature (e.g. normal parenchyma). At least 50 cells/areas should be outlined and saved as TIFs. Note by, these cells/areas should be different than images chosen for creation of algorithm. 2. Upload TIFs into inForm, run algorithm and analyse output. Ensure algorithm can accurately distinguish positive from negative features |

**Supplementary Methods Table S3**. Modified HAI grading: necroinflammatory scores for chronic viral hepatitis (adapted from Ishak et al. 1995 J. of Hepatology). (HAI = hepatic activity index).

| **Category** | **Score** |
| --- | --- |
| **A. Piecemeal Necrosis** |  |
| Absent | 0 |
| Mild (focal, few areas) | 1 |
| Mild/moderate (focal, most portal areas) | 2 |
| Moderate (continuous around <50% of tracts or septa) | 3 |
| Severe (continuous around >50% of tracts or septa) | 4 |
| **B. Confluent Necrosis** |  |
| Absent | 0 |
| Focal confluent necrosis | 1 |
| Zone 3 necrosis in some areas | 2 |
| Zone 3 necrosis in most areas | 3 |
| Zone 3 necrosis + occasional portal-central bridging | 4 |
| Zone 3 necrosis + multiple portal-central bridging | 5 |
| Panacinar or multiacinar necrosis | 6 |
| **C. Focal lyptic necrosis, apoptosis and focal inflammation* (lobular necrosis)** |  |
| Absent | 0 |
| One focus or less per 10x objective | 1 |
| Two to four foci per 10x objective | 2 |
| Five to ten foci per 10x objective | 3 |
| More than ten foci per 10x objective | 4 |
| **D. Portal inflammation** |  |
| None | 0 |
| Mild, some or all portal areas | 1 |
| Moderate, some or all portal areas | 2 |
| Moderate/marked, all portal areas | 3 |
| Marked, all portal areas | 4 |

*Does not include diffuse sinusoidal infiltration by inflammatory cells.

HAI = hepatic activity score. Calculated by obtaining the composite of each histological feature.

**Supplementary Methods Table S4: surrogate measures for Ishak-Knodell components**

| **Component** | **Ishak-Knodell** | **Surrogate InForm Measure** | **Manual component** |
| --- | --- | --- | --- |
| **Piecemeal necrosis** | Degeneration/loss of hepatocytes and associated inflammatory infiltrate within limiting plate. Scores represent the percentage of portal tracts that are affected either focally or continuously | Percentage of CD45^+ve^ cells within limiting plate | Limiting plates must be delineated manually. |
| **Confluent necrosis** | Substantial area of cell death. Scores represent the lobular areas affected; “some” versus “most”, and further includes portal-central bridging in higher scores. | Percentage of lobular necrotic tissue | Lobular areas must be delineated manually |
| **Lobular necrosis** | The number of inflammatory foci present in x10 fields of view | Percentage of lobular CD45^+ve^ inflammatory cells that are present as clusters (foci) | Lobular areas must be delineated manually |
| **Portal inflammation** | Amount of inflammatory cells present in “some” versus “all” portal tracts | Percentage of portal CD45^+ve^ inflammatory cell | Portal areas must be delineated manually. |
| **HAI** | Hepatic Activity Score. Composite of the above scores. | Composite of the above scores | -- |

**Supplementary Methods Table S5. Cohort Information.**

| **Patient ID** | **Sex** | **Notes** | **Scoring System** | **Category & Scores** | |
| --- | --- | --- | --- | --- | --- |
| Hep1 | M | Liver biopsy with severe acute hepatitis and submassive hepatic necrosis.  Aetiology: autoimmune. | Ishak-Knodell | Piecemeal necrosis | 4 |
|  |  |  |  | Confluent necrosis | 5 |
|  |  |  |  | Lobular necrosis^#^ | 3 |
|  |  |  |  | Portal inflammation | 4 |
|  |  |  |  | HAI^$^ | 16 |
| Hep2 | ? |  | Ishak-Knodell | Piecemeal necrosis | 1 |
|  |  |  |  | Confluent necrosis | 0 |
|  |  |  |  | Lobular necrosis^#^ | 0 |
|  |  |  |  | Portal inflammation | 1 |
|  |  |  |  | HAI^$^ | 2 |
| Hep3 | M | Liver biopsy with mild/ moderate acute hepatitis in HIV+, on TB treatment.  Aetiology: most likely drug related | Ishak-Knodell | Piecemeal necrosis | 2 |
|  |  |  |  | Confluent necrosis | 3 |
|  |  |  |  | Lobular necrosis^#^ | 3 |
|  |  |  |  | Portal inflammation | 2 |
|  |  |  |  | HAI^$^ | 10 |
| Hep4 | M | Liver biopsy with severe acute hepatitis and cholestasis.  Aetiology: autoimmune. | Ishak-Knodell | Piecemeal necrosis | 4 |
|  |  |  |  | Confluent necrosis | 4 |
|  |  |  |  | Lobular necrosis^#^ | 4 |
|  |  |  |  | Portal inflammation | 4 |
|  |  |  |  | HAI^$^ | 16 |
| Hep5 | M | Liver biopsy with moderate/severe acute hepatitis and cholestasis.  Aetiology: autoimmune. | Ishak-Knodell | Piecemeal necrosis | 2 |
|  |  |  |  | Confluent necrosis | 2 |
|  |  |  |  | Lobular necrosis^#^ | 2 |
|  |  |  |  | Portal inflammation | 1 |
|  |  |  |  | HAI^$^ | 7 |
| Hep6 | M | Liver biopsy with moderate acute hepatitis and mild cholestasis.  Aetiology: most likely drug related. | Ishak-Knodell | Piecemeal necrosis | 1 |
|  |  |  |  | Confluent necrosis | 0 |
|  |  |  |  | Lobular necrosis^#^ | 1 |
|  |  |  |  | Portal inflammation | 1 |
|  |  |  |  | HAI^$^ | 3 |
| Hep7 | M | Liver biopsy with acute hepatitis, marked parenchymal cholestasis and moderate portal inflammation.  Aetiology: most likely drug related. | Ishak-Knodell | Piecemeal necrosis | 2 |
|  |  |  |  | Confluent necrosis | 4 |
|  |  |  |  | Lobular necrosis^#^ | 1 |
|  |  |  |  | Portal inflammation | 2 |
|  |  |  |  | HAI^$^ | 9 |
| Hep8 | F | Liver biopsy with severe acute hepatitis. | Ishak-Knodell | Piecemeal necrosis | 3 |
|  |  |  |  | Confluent necrosis | 5 |
|  |  |  |  | Lobular necrosis^#^ | 3 |
|  |  |  |  | Portal inflammation | 2 |
|  |  |  |  | HAI^$^ | 13 |
| Hep9 | F | Liver biopsy with moderate acute hepatitis in patient with IBD on mesalazine.  Aetiology: most likely drug related | Ishak-Knodell | Piecemeal necrosis | 2 |
|  |  |  |  | Confluent necrosis | 3 |
|  |  |  |  | Lobular necrosis^#^ | 2 |
|  |  |  |  | Portal inflammation | 2 |
|  |  |  |  | HAI^$^ | 9 |
| Hep10 | F | Liver biopsy with severe acute hepatitis, 6 days post-partum.  Aetiology unknown. | Ishak-Knodell | Piecemeal necrosis | 4 |
|  |  |  |  | Confluent necrosis | 3 |
|  |  |  |  | Lobular necrosis^#^ | 3 |
|  |  |  |  | Portal inflammation | 3 |
|  |  |  |  | HAI^$^ | 13 |
| Hep11 | F | Liver biopsy with severe acute hepatitis.  Aetiology: viral CMV. | Ishak-Knodell | Piecemeal necrosis | 4 |
|  |  |  |  | Confluent necrosis | 3 |
|  |  |  |  | Lobular necrosis^#^ | 3 |
|  |  |  |  | Portal inflammation | 3 |
|  |  |  |  | HAI^$^ | 13 |
| NAFLD1 | ? | Fine needle aspiration. |  |  |  |
| NAFLD2 | M | Liver biopsy with severe steatosis in patient with NAFLD. |  |  |  |
| NAFLD3 | F | Liver biopsy with mild steatosis in patient with NAFLD. |  |  |  |

^#^focal lyptic necrosis, apoptosis and focal inflammation

^$^Hepatic activity index

**Supplementary Methods Table S6: Parameters of Aperio’s positive pixel count algorithm (v9.)**

| **Parameter** | **Input** |
| --- | --- |
| Mark-up compression type | Same as processed image |
| Compression quality | 30 |
| Classified neighbourhood | 0 |
| Classifier | None |
| Class list | Blank |
| Hue value | 0.11 |
| Hue width | 0.5 |
| Colour Saturation Threshold | 0.18 |
| Iwp(High) | 255 |
| Iwp(Low)=Ip(High) | 180 |
| Ip(Low)=Isp(High) | 179 |
| Isp(low) | 0 |
| Inp(high) | 255 |

**Supplementary Methods: pixel counting using Aperio ImageScope**

Liver sections stained with PCK or GCTM-5 immunohistochemically were used for automatic quantitation. The sections were scanned using an Aperio Digital Scanscope XT (Leica) at 40x magnification, and analysed using Aperio ImageScope software (V12.0). The total pixel count of each section was obtained by outlining their edge carefully using the layering tool. Holes in the tissue and lumen of ducts were excluded in the total pixel count. To obtain the area of PCK^+ve^ or GCTM-5^+ve^ cells, the Positive Pixel Count V9 algorithm was used, which is based on colour thresholding. Parameters used are shown in Supplementary Methods Table S6. The final result was expressed as the number of PCK^+ve^ or GCTM-5^+ve^ pixels as a percentage of the total pixel count.
